# Indoor ozone/human chemistry and ventilation strategies

**DOI:** 10.1111/ina.12594

**Published:** 2019-09-15

**Authors:** Christian Mark Salvador, Gabriel Bekö, Charles J. Weschler, Glenn Morrison, Michael Le Breton, Mattias Hallquist, Lars Ekberg, Sarka Langer

**Affiliations:** ^1^ Department of Chemistry and Molecular Biology Atmospheric Sciences University of Göteborg Göteborg Sweden; ^2^ International Centre for Indoor Environment and Energy Department of Civil Engineering Technical University of Denmark Lyngby Denmark; ^3^ Environmental and Occupational Health Sciences Institute Rutgers University Piscataway NJ USA; ^4^ Department of Environmental Sciences and Engineering Gillings School of Global Public Health The University of North Carolina at Chapel Hill Chapel Hill NC USA; ^5^ CIT Energy Management AB Göteborg Sweden; ^6^ Division of Building Services Engineering Department of Architecture and Civil Engineering Chalmers University of Technology Göteborg Sweden; ^7^ IVL Swedish Environmental Research Institute Göteborg Sweden; ^8^ Present address: Volvo Group Trucks and Technology Method and Technical Development Göteborg Sweden

**Keywords:** air exchange rate, indoor environment, oxygenated volatile organic compounds, ozone, squalene, ToF‐CIMS

## Abstract

This study aimed to better understand and quantify the influence of ventilation strategies on occupant‐related indoor air chemistry. The oxidation of human skin oil constituents was studied in a continuously ventilated climate chamber at two air exchange rates (1 h^−1^ and 3 h^−1^) and two initial ozone mixing ratios (30 and 60 ppb). Additional measurements were performed to investigate the effect of intermittent ventilation (“off” followed by “on”). Soiled t‐shirts were used to simulate the presence of occupants. A time‐of‐flight‐chemical ionization mass spectrometer (ToF‐CIMS) in positive mode using protonated water clusters was used to measure the oxygenated reaction products geranyl acetone, 6‐methyl‐5‐hepten‐2‐one (6‐MHO) and 4‐oxopentanal (4‐OPA). The measurement data were used in a series of mass balance models accounting for formation and removal processes. Reactions of ozone with squalene occurring on the surface of the t‐shirts are mass transport limited; ventilation rate has only a small effect on this surface chemistry. Ozone‐squalene reactions on the t‐shirts produced gas‐phase geranyl acetone, which was subsequently removed almost equally by ventilation and further reaction with ozone. About 70% of gas‐phase 6‐MHO was produced in surface reactions on the t‐shirts, the remainder in secondary gas‐phase reactions of ozone with geranyl acetone. 6‐MHO was primarily removed by ventilation, while further reaction with ozone was responsible for about a third of its removal. 4‐OPA was formed primarily on the surfaces of the shirts (~60%); gas‐phase reactions of ozone with geranyl acetone and 6‐MHO accounted for ~30% and ~10%, respectively. 4‐OPA was removed entirely by ventilation. The results from the intermittent ventilation scenarios showed delayed formation of the reaction products and lower product concentrations compared to continuous ventilation.

**Practical Implications**

- In occupied buildings, ventilation strategies do not offer a simple approach for controlling products that may arise from ozone/human chemistry. This reflects the fact that such products are generated from both surface reactions and gas‐phase reactions.
- Surface reactions are relatively insensitive to ventilation rates while the gas‐phase reactions compete with ventilation.
- Exposure to noxious products of ozone/human chemistry can be reduced by decreasing ventilation during periods with high outdoor ozone levels. When buildings are unoccupied (eg, overnight or on weekends), decreasing or stopping ventilation reduces indoor ozone chemistry and surface accumulation of oxidation products.
- Results from this study can serve as inputs to predictive models of ozone/human interaction in indoor environments.

## 1 INTRODUCTION

The importance of indoor air chemistry has been increasingly recognized over the past decades.^1^, ^2^, ^3^, ^4^ Building materials, furnishings and carpeting, cleaning products, personal care products, human activities, as well as outdoor air have been considered major sources of indoor air pollutants. During the past decade, there has been growing recognition that humans themselves considerably contribute to indoor air pollution and impact indoor air chemistry.^5^, ^6^

Squalene constitutes approximately 12% (by weight) of human skin lipids; other constituents include fatty acids, glycerides, wax esters, ceramides, and cholesterol esters.^7^ Having six carbon‐carbon double bonds in its structure, squalene serves as a natural antioxidant to protect our skin from atmospheric oxidants such as ozone (O_3_), which reacts with the unsaturated sites of the molecule. Squalene is responsible for about half of all unsaturations available in skin lipids,^8^ and given the slight differences in reaction probabilities between unsaturations in squalene and fatty acids and their esters, it is responsible for approximately half the ozone uptake.

Ozone is the most abundant oxidant in the indoor environment. Its major source indoors is outdoor air, entering the buildings via ventilation and infiltration. The indoor‐to‐outdoor (I/O) concentration ratio of ozone is usually between 0.1 and 0.7, depending on the building air exchange rate (AER) and the properties of the exposed surfaces.^9^

Ozonolysis of surface‐bound squalene was first investigated on Mediterranean vegetation.^10^ The complex reaction of squalene with O_3_ produces a large spectrum of oxygenated volatile species, including acetone, geranyl acetone (GA), 6‐methyl‐5‐hepten‐2‐one (6‐MHO) and 4‐oxopentanal (4‐OPA). These were reported in a simulated aircraft cabin containing soiled t‐shirts in 2005^11^ and in the same simulated cabin with human “passengers” in 2007.^12^ Further details of ozone/human occupant chemistry were reported by Wisthaler and Weschler^13^ in a controlled experiment conducted in a climate chamber representing a simulated office.

In subsequent studies, 4‐OPA was shown to be a sensory irritant and capable of inducing inflammatory cytokine expression in vitro*.* 14, 15, 16 The derived human reference value for sensory irritation by 6‐MHO is 0.3 ppm, and the reference values for airflow limitation are 0.03 and 0.5 ppm for 4‐OPA and 6‐MHO, respectively.^17^

Other volatile secondary products of this reaction include 4‐methyl‐8‐oxo‐4‐nonenal (4‐MON), 4‐methyl‐4‐octene‐1,8‐dial (4‐MOD), 1,4‐butanedial and carboxylic acids such as 4‐oxo‐butanoic acid and 4‐oxopentanoic (levulinic) acid. In addition to the volatile products, the ozonolysis of squalene also generates a number of long‐chained polyunsaturated aldehydes and carboxylic acids such as C27‐pentaenal, C22‐tetraenal, C17‐trienal, C27‐pentaenoic acid, C22‐tetraenoic acid, C17‐trienoic acid, that remain bound to the surface. Surface reactions remove ozone from indoor environments more effectively than gas‐phase reactions.^18^ The human surface has been shown to be very effective at removing ozone.^12^, ^13^, ^19^

The oxygenated and highly oxygenated products of squalene ozonolysis are difficult to measure in indoor air by conventional techniques such as sampling on adsorbent tubes followed by thermal desorption into a gas chromatograph with, for example, mass selective detector (TD/GC‐MS). These compounds are often thermally unstable and undergo decomposition in the thermal desorption stage. Even if some of these “stealth compounds” may be suitable for the classical TD/GC‐MS analysis, the time resolution is in minutes to hours due to sampling on the adsorbents.

Development of monitoring instruments with time resolution in minutes to seconds, based on mass spectrometry, has enabled the determination and concentration‐time profiling of a range of volatile organic compounds in air. Proton transfer reaction mass spectrometer (PTR‐MS) was used, for example, in the above‐mentioned simulated aircraft cabin^11^, ^12^ and office experiments.^13^ Other studies looked at ozone removal and product formation on clothes,^20^ human hair^8^, pieces of clothes soaked with methanol extracts of human skin lipids,^21^ on the heterogeneous mechanism of squalene/ozone reaction,^22^ and on modeling of the processes with relevance to indoor environments.^18^, ^19^ Building ventilation is used to control the thermal environment and the concentrations of indoor air pollutants. Experimental and modeling studies have demonstrated the impact of ventilation on indoor chemistry and product formation.^18^, ^23^, ^24^

The air exchange rate (AER) has only a small impact on chemical reactions that occur on indoor surfaces. However, for indoor reactions in the gas‐phase, AER determines the time available for reactions to occur. While higher AER shortens the time available for reactions, it increases indoor ozone concentrations relative to their outdoor level. That is, low AER transports less ozone from outdoors to indoors, but allows more time for chemistry.^1^ Energy saving measures have led to the implementation of alternative ventilation strategies, such as intermittent ventilation, where the ventilation system operates with minimal or no airflow overnight. Different ventilation strategies are anticipated to affect chemical transformations in indoor air differently.

The objective of this study was to investigate the effect of continuous and intermittent ventilation strategies on occupant‐related ozone chemistry. Dynamic and steady‐state mixing ratios of oxygenated products of skin oil ozonolysis were measured. To gain a better understanding of simultaneous formation and removal processes, product yields were derived by fitting a series of mass balance equations to the data.

## 2 METHODS

### 2.1 Climate chamber

The experiments were performed in a 30 m^3^ stainless steel climate chamber. In order to remove reactive compounds from the surfaces, the chamber was treated with 375 ppb of ozone during a period of 72 hours prior to the first experiment. During the experiments, the temperature in the chamber was maintained at 23°C. The relative humidity (RH) was not controlled, but was relatively constant around 35%. The air exchange rate (AER) was set to 1 or 3 h^−1^ (Table 1). It was measured regularly during the experimental month with an Innova 1312 Photoacoustic Multi‐gas Monitor (LumaSense Technologies A/S, Ballerup, Denmark) using Freon^®^ 134a as tracer gas. The chamber's HVAC system contained a particle filter and an activated carbon filter. The background ozone mixing ratio in the chamber was <2 ppb.

**Table 1:** Experimental matrix, for all Conditions except 2, t‐shirts placed in chamber at t = 0 h

Condition*	AER (h^−1^)	ozone (ppb)	Note
1	1	0	Reference condition—with t‐shirts
2	1	60	Reference condition—without t‐shirts
3	1	30	
4	1	60	Base day for Condition 8**
5	3	30	
6	3	60	
7	1	60	Base day for Condition 9**
8	1	60	Ventilation and O_3_ generation started at t = 0 h
9	1	60	Ventilation and O_3_ generation started at t = −2 h

* Condition 8 followed Condition 4, Condition 9 followed Condition 7. Conditions 4 and 7 were identical. Due to analytical issues, data from Condition 4 were unavailable. Data from Condition 7 were not consistent with data obtained in the other experiments—see ‘Limitations of the Study’.

** O_3_ generation stopped, t‐shirts removed and AER lowered to 0.15 h^−1^ at the end of experiment.

Ozone was generated in the HVAC system downstream of the activated carbon filter by delivering air through a Jelight 600 UV ozone generator (Jelight Company Inc). The ozone generation rate was controlled by changing the fraction of the UV lamp that was exposed. The amount of air drawn through the ozone generator (0.5 L min^−1^) was controlled with a flow meter. Ozone was continuously monitored with an Environics Series 300 UV absorption ozone monitor with a time resolution of 1 minute and accuracy of ±2 ppb (Environics Inc). Temperature and relative humidity were continuously monitored with 1‐minute time resolution using Wöhler CDL 210 sensors (Wöhler Technik GmbH).

### 2.2 Experimental conditions

Human occupancy was simulated using recently worn t‐shirts. Plain, white, cotton t‐shirts (exposed surface area of one t‐shirt is approximately 0.85 m^2^) were washed at 60°C, tumble dried, and stored in zip‐locked plastic bags before the experiments. Before a measurement, the same four persons were asked to wear fresh t‐shirts overnight and come to the laboratory in them without showering.^11^ The four t‐shirts were then turned inside out and stretched over the backs of wire mesh chairs in the chamber. Two mixing fans were operating in the chamber.

Two sets of experiments were performed (Table 1). The first set (Conditions 1‐7) examined the effect of two air exchange rates (1 and 3 h^−1^) and three levels of ozone (<2, 30, and 60 ppb) in the chamber on occupant‐related indoor air chemistry. The experimental conditions were established the evening before, insuring sufficient time to reach steady‐state ozone mixing ratio in the chamber before the commencement of the experiment in the morning (“continuous ventilation”). At the end of each measurement day, the chamber was flushed with fresh air (AER = 20 h^−1^ for 20 minute) before setting the conditions for the next experimental day (except after Condition 4; see below). The overnight ozonation of the empty chamber also served as cleaning/passivation of the chamber walls for the next experiment. The second set of experiments (Conditions 8‐9) examined the impact of “intermittent ventilation.” These experiments included two consecutive days of measurements. An intermittent ventilation scheme was simulated by stopping the ozone generation and lowering the AER to approximately 0.15 h^−1^ (simulating infiltration) at the end of the first day of measurements. Conditions 4 and 7 (identical) served as base days in order to have identical starting points for Conditions 8 and 9. The following morning, ozone generation was started and the AER was increased to 1 h^−1^ either at the time when the shirts were placed in the chamber (t = 0 hour, Condition 8) or 2 hours before (t = −2 hour, Condition 9).

### 2.3 ToF‐CIMS measurement

The Aerodyne ToF‐CIMS was utilized for the measurement of gas‐phase species produced in the chamber. The work described in this paper used the ToF‐CIMS in positive mode with protonated water clusters as reagent ions. A detailed description of the instrument can be found elsewhere.^25^

The sample air was brought into the ion molecule region (IMR) at two standard liters per minute (SLM) through a 10 mm outer diameter and 2‐meter‐long inlet. Nitrogen (99.9% purity) flowed at 2.2 LPM through a fritted glass bubbler filled with deionized water to create a high concentration of water vapor. It was subsequently ionized by a polonium source to produce the (H_2_O)_n_H^+^ ions via the addition of a hydrogen atom which then entered the IMR orthogonal to the sample flow. The ionization then proceeded via the following reaction:

$${\left(H_{2}O\right)}_{n}H^{+}+M\to {\text{n(H}}_{2}\text{O)}+{\text{MH}}^{+}$$

The IMR pressure was maintained at 150 mbar and a temperature of 50°C. The pressure of the collisional dissociation chamber (CDC), the chamber after the IMR and containing the first quadrupole (small segmented quadrupole), was held at 2 mbar. The field strengths within the ToF‐CIMS were tuned to be as weak as possible to reduce fragmentation of product ions. Within this setup, we observed the (H_2_O)H^+^, (H_2_O)_2_H^+^ and the (H_2_O)_3_H^+^ clusters with the dimer cluster contributing to 65% of the total ionization cluster counts. This differs from the standard operation of a PTR‐MS where the (H_2_O)H^+^ ion is the dominant ionization signal, likely due to the nascence of a drift tube and therefore lower control of ion energy within the system. We therefore optimized the (H_2_O)H^+^ counts to increase sensitivity and normalize the spectra to the sum of all three cluster ionization ions. The sensitivity of the CIMS also depends on the distribution of reagent ions, which is indicated by the ratio of the dimer ((H2O)_2_H^+^) to its monomer ((H_2_O)H^+^). This ratio was maintained throughout the campaign with less than 10% variation.

Data were acquired at 1s time resolution and averaged to 1 minute for data analysis using the software Tofware 3.1.1 (Aerodyne Inc). Post‐campaign mass calibration of the spectra was conducted using four constant peaks on the spectra; (H_2_O)H^+^, (H_2_O)_2_H^+^, (H_2_O)_3_H^+^, and C_5_H_9_O_2_^+^, enabling an accuracy of 5 ppm for peak identification. The average spectral mass resolution (m/Δm) was 3500.

Permeation sources held at constant temperature were utilized for calibration. Compound standards for acetone, 6‐MHO, and geranyl acetone (GA) were purchased from Sigma‐Aldrich and 5 mL was placed in a small vial with an orifice in the lid. An N_2_ flow of 2.5 SLM was passed through a large glass vial holding the permeation source and then partial flow was sampled by the TOF‐CIMS with the excess being evacuated to an exhaust. The mixing ratio in the flow was calculated using a mass loss approach (see Supporting Information for a more detailed description). The sensitivities for acetone, 6‐MHO, and geranyl acetone were calculated to be 12.77, 4069, and 2785 ion counts per ppb, respectively. We quantified 4‐OPA using the sensitivity factor of 6‐MHO, ensuring that humidity normalization and total ion count signal were constant for each experiment.

### 2.4 Experimental procedure

Each experiment was initiated by placing four t‐shirts in the chamber. The pressurized chamber door was open for less than 3 seconds, a person entered and the door was closed. After placing the t‐shirts on the chairs, the chamber door was open again as the person was leaving. Opening the chamber door may have affected the condition in the chamber. However, no substantial impact on the ozone mixing ratio was observed; ozone mixing ratio decays followed from the beginning the pattern expected after introducing the soiled t‐shirts (see Figure 2). The experiment continued for 3 hours with measurements of ozone and the organic compounds with the ToF‐CIMS. This time interval was judged to be sufficient for the system to reach at least 95% steady‐state mixing ratios of ozone and primary reaction products at both target air exchange rates. During this phase, concentration‐time profiles of ozone and the primary and secondary squalene/ozone reaction products were monitored with 1‐minute time resolution. After 90 minutes, desorption of particles collected during the preceding 30 minutes of gas‐phase measurement was performed using the Filter Inlet for Gases and AEROsols (FIGAERO).^26^ We plan to present results from the particle measurements in a future article. After the FIGAERO desorption process, it took approximately 10 minutes for the measured mixing ratios of gas‐phase species to reach those values immediately prior to particle desorption. After the experiment, the t‐shirts were removed from the chamber and the chamber was flushed with outdoor air (20 h^−1^ for 20 minutes).

### 2.5 Mass balance model

**Figure 1:**
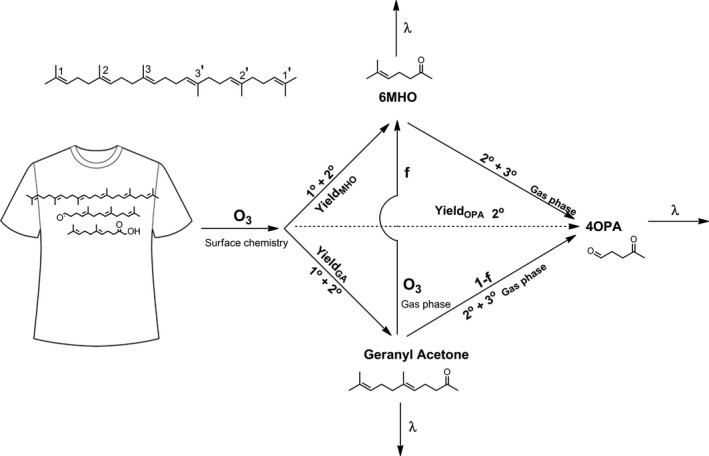
Visualization of the formation and loss of geranyl acetone, 6‐MHO, and 4‐OPA (f – branching ratio; 1°, 2°, 3° – primary, secondary, tertiary reaction; λ – air exchange rate)

A series of mass balance models were used to describe the steady‐state conditions of ozone and the oxygenated products of ozone‐squalene reaction inside the chamber during the continuous measurements (Conditions 3, 5‐7). The models are described below and visualized in Figure 1.

#### 2.5.1 Ozone

(1) $$\frac{d[O_{3}]}{\text{dt}}=\text{Emission rate}-\lambda \left[O_{3}\right]-k_{\text{sur}}\left[O_{3}\right]-k_{6\text{MHO}}\left[O_{3}\right]\left[6\text{MHO}\right]-k_{\text{GA}}\left[O_{3}\right]\left[\text{GA}\right]$$

Equation (1) can be rewritten for steady‐state condition (d[O_3_]/dt = 0) as:

(2) $$\text{Emission Rate}=\lambda {\left[O_{3}\right]}_{\text{ss}}+k_{\text{sur}}{\left[O_{3}\right]}_{\text{ss}}+k_{6\text{MHO}}{\left[O_{3}\right]}_{\text{ss}}{\left[6\text{MHO}\right]}_{\text{ss}}+k_{\text{GA}}{\left[O_{3}\right]}_{\text{ss}}{\left[\text{GA}\right]}_{\text{ss}}$$

where emission rate (ppb h^−1^) is the ozone generation rate in the chamber, λ (h^−1^) is the air exchange rate, *k*_sur_ (h^−1^) is the overall, first‐order ozone removal rate due to reactions with the stainless steel chamber surfaces and the soiled t‐shirts, *k*_6MHO_ and *k*_GA_ (ppb^−1^ h^−1^) are the gas‐phase, second‐order reaction rate coefficients for 6‐MHO and GA with ozone, respectively, [O_3_]_ss_, [6MHO]_ss_, and [GA]_ss_ are the steady‐state mixing ratios of ozone, 6‐MHO, and geranyl acetone, respectively (ppb). *k*_sur_ was based on steady‐state ozone conditions, where [O_3_]_ss_ = O_3_ emission rate/sum of sinks ~O_3_ emission rate/(λ + k_sur_). The average value of *k*_sur_ was estimated to be (1.37 ± 0.11) h^−1^. The calculation is shown in Supporting Information.

The ozone removal rate due to its loss on the chamber walls was calculated from the decay of ozone in the empty chamber at the end of the experiments for Conditions 4 and 7, when ozone generation was stopped and the AER lowered to 0.15 h^−1^. The measured value of k_chamber_ was 0.16 h^−1^ and 0.15 h^−1^ in these two experiments and is consistent with ozone loss rates previously reported^27^, ^28^, ^29^, ^30^ in empty stainless steel chambers (between 0.06 and 0.35 h^−1^). In the following Equations (1), (2), (3), (4), (5), (6), *k*_shirt_, the first‐order ozone removal rate coefficient by the t‐shirts, was calculated as *k*_shirt_ = *k*_sur_ − k_chamber_ = (1.2 ± 0.1) h^−1^. The error for *k*_sur_ was estimated to be 8%, and we used this value to calculate the uncertainty of *k*_shirt_.

All parameters in the equations are known, measured in these experiments or estimated based on the measurements, with the exception of *k*_GA_. Similar to Lakey et al,^18^ we have chosen to use 0.068 ppb^−1^ h^−1^ for *k*_GA_; this is twice the value of *k*_6MHO_ = 0.034 ppb^−1^ h^−1^ from Fruekilde et al,^10^ consistent with the two double bonds in the geranyl acetone molecule and given the molecular similarity of GA to 6‐MHO.

#### 2.5.2 Geranyl acetone (GA)

Geranyl acetone is primarily produced through surface reaction of ozone with squalene on the t‐shirts when ozone adds to double bonds 3 or 3′ in the squalene molecule (Figure 1). Additional geranyl acetone is produced through secondary surface reactions between ozone and the long‐chained polyunsaturated aldehydes and carboxylic acids (C27‐pentaenal, C22‐tetraenal, C17‐trienal, C27‐pentaenoic acid, C22‐tetraenoic acid, and C17‐trienoic acid) remaining on the shirts.

(3) $$\frac{d[\text{GA}]}{\text{dt}}=k_{\text{shirt}}{\text{Yield}}_{\text{GA}}\left[O_{3}\right]-\lambda \left[\text{GA}\right]-k_{\text{GA}}\left[O_{3}\right]\left[\text{GA}\right]$$

Equation (3) can be rewritten for steady‐state condition (d[GA]/dt = 0) as:

(4) $$k_{\text{shirt}}{\text{Yield}}_{\text{GA}}{\left[O_{3}\right]}_{\text{ss}}=\lambda {\left[\text{GA}\right]}_{\text{ss}}+k_{\text{GA}}{\left[O_{3}\right]}_{\text{ss}}{\left[\text{GA}\right]}_{\text{ss}}$$

where Yield_GA_ (unitless) is the yield of geranyl acetone from the primary and the secondary reactions of ozone on the shirts.

#### 2.5.3 6‐methyl‐5‐hepten‐2‐one (6‐MHO)

6‐MHO is formed as a primary product of ozone/squalene reactions on the surface of the t‐shirts when ozone adds to the double bonds 2 or 2′ in the squalene molecule (Figure 1). It can also be formed through secondary reactions both on the t‐shirts and in the gas‐phase as ozone reacts with geranyl acetone.

(5) $$\frac{d[6MHO]}{\text{dt}}=k_{\text{shirt}}{\text{Yield}}_{6\text{MHO}}\left[O_{3}\right]+k_{\text{GA}}f_{6\text{MHO}}\left[O_{3}\right]\left[\text{GA}\right]-\lambda \left[6\text{MHO}\right]-k_{6\text{MHO}}\left[O_{3}\right]\left[6\text{MHO}\right]$$

Equation (5) can be rewritten for steady‐state condition (d[6MHO]/dt = 0) as:

(6) $$k_{\text{shirt}}{\text{Yield}}_{6\text{MHO}}{\left[O_{3}\right]}_{\text{ss}}+k_{\text{GA}}f_{6\text{MHO}}{\left[O_{3}\right]}_{\text{ss}}{\left[\text{GA}\right]}_{\text{ss}}=\lambda {\left[6\text{MHO}\right]}_{\text{ss}}+k_{6\text{MHO}}{\left[O_{3}\right]}_{\text{ss}}{\left[6\text{MHO}\right]}_{\text{ss}}$$

where Yield_6MHO_ (unitless) is the yield of 6‐MHO from primary (squalene) and secondary (C27‐pentaenal, C22‐tetraenal, C17‐trienal, C27‐pentaenoic acid, C22‐tetraenoic acid, C17‐trienoic acid, and geranyl acetone) reactions of ozone on the t‐shirts, and f_6MHO_ is the fraction of the gas‐phase reaction of ozone with geranyl acetone that produces 6‐MHO (branching ratio). The branching ratios for 6‐MHO and 4‐OPA from the gas‐phase reaction of ozone and GA are unknown; we have set the value of f_6MHO_ to 0.3 in accordance with the findings described by Grosjean and Grosjean (1997).^31^ The sum of formation yields of primary carbonyls is close to one, and the branching ratio favors the primary carbonyl with the more substituted associated biradical.

#### 2.5.4 4‐oxopentanal (4‐OPA)

4‐OPA is formed as a secondary product from ozone reacting with C27‐pentaenal and C22‐tetraenal on shirts, as a secondary product of ozone reacting with geranyl acetone in the gas‐phase, and as secondary or tertiary product from ozone reacting with 6‐MHO in the gas‐phase (Figure 1).

(7) $$\frac{d[4\text{OPA}]}{\text{dt}}=k_{\text{shirt}}{\text{Yield}}_{4\text{OPA}}\left[O_{3}\right]+k_{6\text{MHO}}\left[O_{3}\right]\left[6\text{MHO}\right]+k_{\text{GA}}f_{\text{4OPA}}\left[O_{3}\right]\left[\text{GA}\right]-\lambda \left[4\text{OPA}\right]$$

Equation (7) can be rewritten for steady‐state condition (d[4OPA]/dt = 0) as:

(8) $$k_{\text{shirt}}{\text{Yield}}_{4\text{OPA}}{\left[O_{3}\right]}_{\text{ss}}+k_{\text{MHO}}{\left[O_{3}\right]}_{\text{ss}}{\left[6\text{MHO}\right]}_{\text{ss}}+k_{\text{GA}}f_{\text{OPA}}{\left[O_{3}\right]}_{\text{ss}}{\left[\text{GA}\right]}_{\text{ss}}=\lambda {\left[4\text{OPA}\right]}_{\text{ss}}$$

where Yield_4OPA_ (unitless) is the yield of 4‐OPA from ozone reactions with products of squalene ozonolysis on the t‐shirts (ie, C27‐pentaenal, C22‐tetraenal, and geranyl acetone). *f*_OPA_ is the fraction of gas‐phase reaction of ozone with geranyl acetone that produces 4‐OPA. Given that 0.3 was used for *f*_6MHO_, we set *f*_4OPA_ = 0.7 (assuming that the sum of the branching ratios is unity). The tertiary formation of 4‐OPA from 6‐MHO is not treated in the model.

Yield_6MHO_, Yield_GA_, and Yield_4OPA_ were adjusted in the model calculations for Conditions 3, 5, 6, and 7 to obtain source‐to‐sink ratios near unity for 6‐MHO, geranyl acetone, and 4‐OPA (see Section 3.2).

## 3 RESULTS AND DISCUSSION

### 3.1 Ozone mixing ratio

The rate at which ozone was removed by the stainless steel chamber surfaces (*k*_chamber_, 0.15 h^−1^) was around 10% of the removal by all surfaces when four soiled t‐shirts were present (*k*_sur_, 1.37 h^−1^). Table 2 lists the measured initial and steady‐state mixing ratios of ozone for all Conditions. No significant change in ozone mixing ratio was observed during the measurement period for Condition 2, in which no t‐shirts were present in the chamber. The steady‐state mixing ratios of ozone were between 12 and 37 ppb when t‐shirts were present. The highest value was observed for Condition 6 with an initial ozone mixing ratio of 54 ppb and an elevated ventilation rate (3 h^−1^). At identical initial ozone mixing ratios, the ozone generation rate is three times higher at AER 3 h^−1^ compared to AER 1 h^−1^. Although low, the measured mixing ratios of the ozone‐squalene reaction products for Condition 1 (soiled t‐shirts, no ozone) indicate the presence of ozone‐initiated reactions on the surfaces of the t‐shirts prior to the experiments.

**Table 2:** Mixing ratios of ozone and major reaction products

Condition	AER (h^−1^)	Target Ozone (ppb)	Target Emission Rate (ppb h^−1^)	Ozone concentration (ppb)		VOC concentration (ppb)*		
				Initial	Steady‐state	GA	6‐MHO	4‐OPA
1	1	0	0	<2.0	<2.0	0.16	0.04	0.06
2**	1	60	60	58	58	ND	ND	ND
3	1	30	30	31	12	1.1	0.65	1.9
4	1	60	60	62	28	NA	NA	NA
5	3	30	90	28	19	0.77	0.27	0.69
6	3	60	180	54	37	0.93	0.58	2.2
7***	1	60	60	63	28	0.29	0.32	2.0
8	1	60	60	0	28	0.083	0.097	0.37
9	1	60	60	53	26	0.027	0.055	0.32

Note

NA, Not available; ND, Not detected.

* The average of the last 40 min for each experiment were used as the steady‐state mixing ratios of VOCs. The mixing ratios were corrected for chamber background.

** No soiled t‐shirts were in the chamber during this experiment.

*** See discussion of Condition 7 in ‘Limitations of the Study’*.*

**Figure 2:**
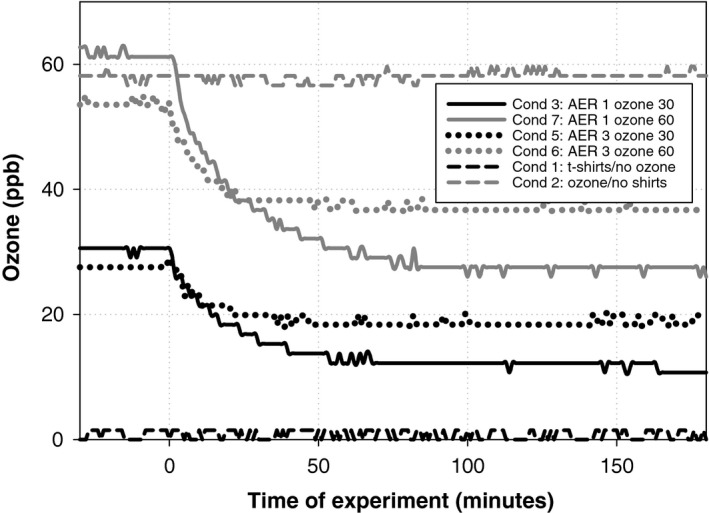
Time profiles of ozone concentration during continuous ventilation experiments (Conditions 1, 2, 3, 5, 6, and 7). T‐shirts were placed in the chamber at t = 0

Figure 2 shows the time series of ozone mixing ratios during continuous ventilation experiments (Conditions 1, 2, 3, 5, 6 and 7). Steady‐state mixing ratios for ozone were achieved 30‐80 minutes after the placement of t‐shirts in the chamber. It is apparent in the figure that it took substantially longer to reach steady‐state at an AER of 1 h^−1^ compared to an AER of 3 h^−1^.

Table 3 presents the parameters in the model that addresses steady‐state ozone mixing ratios. The measured steady‐state ozone mixing ratios fit well with the known ozone generation rates and the identified sinks (source‐to‐sink ratios close to unity; Table 3). Ozone removal rates at steady‐state were dominated by AER (λ) and the removal by reactions on the surfaces of the chamber walls and the t‐shirts (*k*_sur_) (Equation (2); Table 3). Air exchange was responsible for ~40% of the ozone removal rate at 1 h^−1^ and for ~70% at 3 h^−1^. The sum of air exchange rate and ozone removal rate by the surfaces accounted for more than 96% of the loss of ozone in the chamber under all conditions. Gas‐phase reactions with GA and 6‐MHO had a negligible impact on ozone loss compared to ventilation and surface removal. The time to steady‐state for each Condition is consistent with the ozone loss rate being dominated by ventilation and surface removal (λ + k_sur_).

**Table 3:** Model parameters for ozone mass balance (Equation (2))

Condition AER, ozone (h^−1^, ppb)	Source term	Sink terms				Source/sink ratio
	True emission rate (ppb h^−1^)	λ[O_3_]_ss_ (ppb h^−1^)	*k*_sur_[O_3_]_ss_ (ppb h^−1^)	*k*_6MHO_[O_3_]_ss_[MHO]_ss_ (ppb h^−1^)	*k*_GA_[O_3_]_ss_[GA]_ss_ (ppb h^−1^)	
1, 30	31	12	17	0.27	0.90	1.0
1, 60	62	28	38	0.30	0.55	0.93
3, 30	83	57	26	0.17	0.99	0.99
3, 60	161	110	50	0.73	2.33	0.98

Note

The numbers in the column labeled *Condition* refer to the AER (h^−1^) and the ozone mixing ratio (ppb).

The worn t‐shirts accounted for approximately 90% of the removal of ozone by all surfaces (*k*_shirt_/*k*_sur_ = 1.22 h^−1^/1.37 h^−1^ = 0.89). The rate of ozone removal to a highly reactive surface is limited primarily by the resistance to mass transport across the boundary layer of air adjacent to the surface.^32^ This may be the case for the t‐shirts (*k*_shirt_), where the transport‐limited ozone flux controls the maximum rate of reactive uptake of ozone with skin lipids. This is supported by the observation that, from the introduction of the shirts into the chamber until the end of the measurements, ozone consumption occurred at a nearly constant rate. We also observed that *k*_shirt_ varied very little from condition to condition. Given the surface area of the four t‐shirts (3.4 m^2^), the volume of the chamber (30 m^3^), and the value of *k*_shirt_ (1.22 h^−1^), the calculated deposition velocity for ozone to the t‐shirts is 10.8 m h^−1^. This is slightly higher than previously reported ozone/t‐shirt deposition velocities, perhaps reflecting the presence of two mixing fans in the chamber. Tamas et al (2006)^33^ measured deposition velocities of 6.8 and 9.7 m h^−1^ under two sets of experimental conditions, while Rai et al (2014)^34^ measured deposition velocities ranging from 5.4 to 10.4 m h^−1^ in a series of chamber experiments. A recent computational fluid dynamics modeling study^35^ calculates ozone deposition velocities to human surfaces of 8‐9 m h^−1^ at an AER of 3 h^−1^.

The assumption that *k*_shirt_ is constant during the course of an experiment (approx. 3 hours in total) is supported by the observation that the ozone mixing ratio does not vary, within the ±2 ppb accuracy of the measurement, during the final 100 minutes of an experiment (see Figure 2). However, it should be noted that the assumption that the ozone mixing ratio is at steady‐state (ss) is not necessarily valid for prolonged periods of time. After reaching its lowest mixing ratio following the placement of the t‐shirts in the chamber, at some point the ozone mixing ratio begins to slowly increase as squalene and the unsaturated reaction products on the surface of the t‐shirts become depleted. Thus, *k*_shirt_ is not constant over a long‐term (tens of hours) measurement. For example, in a companion measurement of ozone mixing ratio over a period of 45 hours the ozone level rapidly decreased from 63 to 26 ppb (41%) after placing 4 soiled t‐shirts in the chamber and then slowly increased to 41 ppb (64%) after 45 hours (see Supporting Information). With a single soiled t‐shirt in the chamber, it took ~90 hours to reach the initial ozone level established in the chamber before introducing the t‐shirt (data not shown).

**Figure 3:**
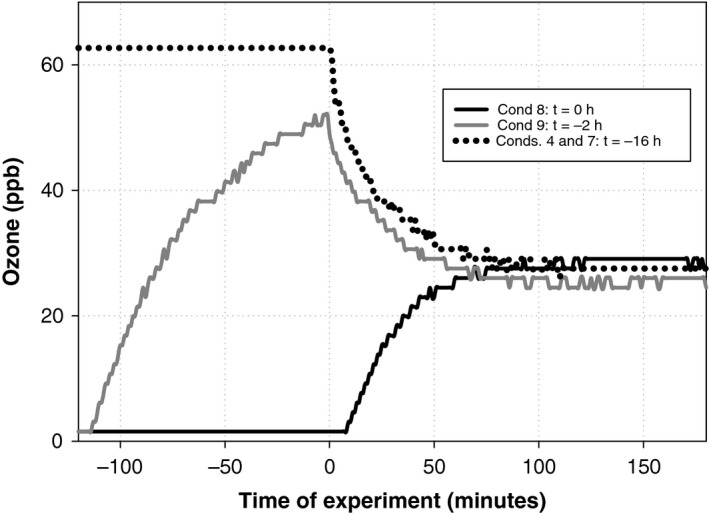
Time profiles of ozone concentration under conditions with intermittent ventilation (Conditions 8 (t = 0 h) and 9 (t = −2 h)). Data from the corresponding continuous ventilation experiment (t = −16, Conditions 4 and 7, 60 ppb ozone, 1 h^−1^ AER) are included for reference. T‐shirts were placed in the chamber at t = 0

Figure 3 compares the time series of ozone mixing ratios for the intermittent ventilation conditions with the corresponding continuous ventilation condition (Conditions 4 and 7 (identical), AER 1 h^−1^, 60 ppb ozone). In Conditions 4 and 7 (continuous ventilation, t = −16 hours) and in Condition 9 (intermittent ventilation, t = −2 hours), steady‐state ozone mixing ratio was approached from an “ozone rich” state. In Condition 8 (intermittent ventilation, t = 0 hour), ozone approached steady‐state from an “ozone poor” state. All three conditions reached similar steady‐state mixing ratios about 60 minutes after placing the t‐shirts in the chamber at time t = 0 hour. This is consistent with the ozone removal rate coefficient for the soiled t‐shirts being determined primarily by mass transport across the boundary layer of air adjacent to the surface of the shirt as opposed to reactions on the surfaces of the shirts. However, by the time steady‐state had been achieved, substantially less surface chemistry had occurred during Condition 8 than during Conditions 4, 7, and 9. Between 0 and 75 minutes, the ozone flux to the shirts for Condition 8 was 46% of the flux for Conditions 4 and 7, while the ozone flux for Condition 9 was 89% of that for Conditions 4 and 7.

### 3.2 Oxygenated VOCs

**Figure 4:**
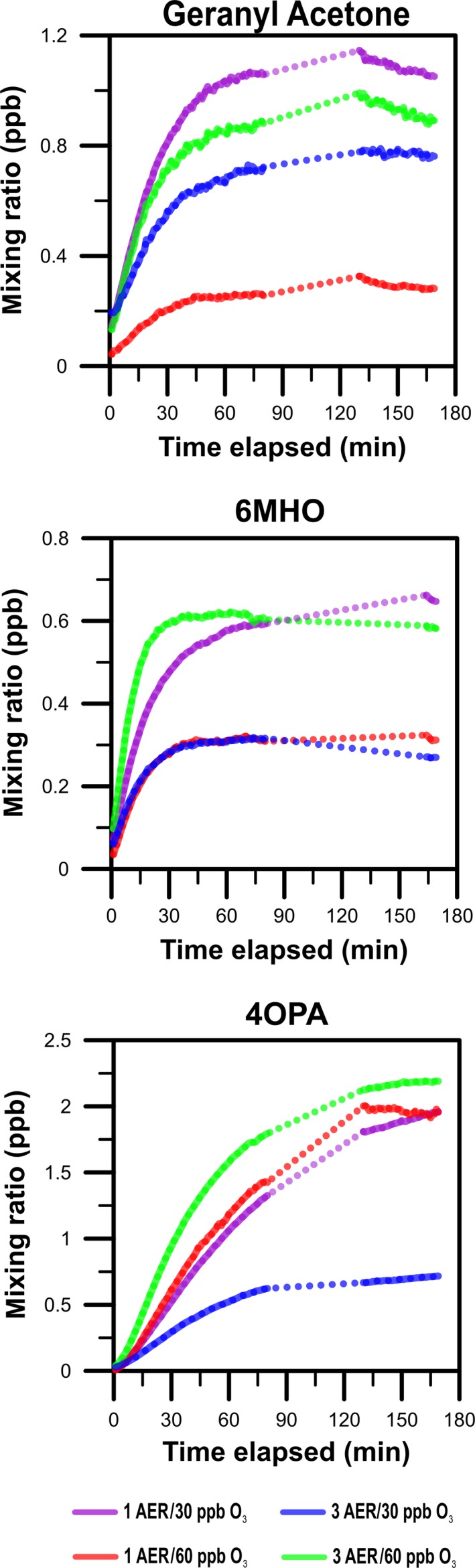
Time series of geranyl acetone, 6‐MHO, and 4‐OPA for different target ozone concentrations and AERs during experiments with continuous ventilation. The gap in the time series is due to thermal desorption of aerosols and the subsequent memory effect

Figure 4 shows the time series for geranyl acetone, 6‐methyl‐5‐hepten‐2‐one, and 4‐oxopentanal, three major volatile oxygenated products of squalene ozonolysis, from the experiments with continuous ventilation. 1,4‐butanedial, 4‐MON, and 4‐MOD were also observed in these experiments. 1,4‐butanedial was not further analyzed due to its low and unreliable signals during the measurements. Time series plots for 4‐MON and 4‐MOD are presented in the Supporting Information (Figure S3). These two compounds have not been included in the mass balance models since doing so would require extensive assumptions. Secondary products such as 4‐OPA, 4‐MON, and 4‐MOD showcase the different influence of air exchange rates on gas‐phase and surface reactions for secondary products. The importance of gas‐phase chemistry follows the order 4‐OPA > 4‐MON >> 4‐MOD. The results shown in Figure S3 are consistent with this order, which is explained by the precursors for these secondary products. 4‐OPA is formed from 6‐MHO and geranyl acetone (primarily in the gas‐phase) and from C27‐pentaenal and C22‐tetraenal (primarily on surfaces); 4‐MON is formed from geranyl acetone (primarily in the gas‐phase) and from C27‐pentaenal (primarily on surfaces); 4‐MOD is formed from C22‐tetraenal and C‐17‐trienal (almost exclusively on surfaces).

Table 2 lists the average steady‐state mixing ratios for geranyl acetone, 6‐MHO, and 4‐OPA (see Supporting Information for the rationale behind the steady‐state assumptions). The values are approximately steady‐state except for Condition 3, where 4‐OPA did not reach steady‐state within the three‐hour measurement. However, its rate of change in mixing ratio during the last 40 minutes was small compared to the first 75 minutes of the experiment. Depending on the Condition, the average mixing ratio for the last 40 minutes was between 0.29 and 1.1 ppb for geranyl acetone, between 0.27 and 0.65 ppb for 6‐MHO, and between 0.69 and 2.2 ppb for 4‐OPA.

Table 4 shows steady‐state model parameters for geranyl acetone. The yield (Yield_GA_), which is that fraction of ozone removed by the shirts that produces geranyl acetone, has been adjusted to produce source‐to‐sink ratios near unity. The estimated effective yield for geranyl acetone was close to 12% for Conditions 3, 5, and 6, while it was only 2.5% for Condition 7.

**Table 4:** Steady‐state model parameters for geranyl acetone

Condition AER, ozone (h^−1^, ppb)	Yield_GA_ (‐)	Source term	Sink terms		Source/sink ratio
		k_shirt_yield_GA_[O_3_]_ss_ (ppb h^−1^)	λ[GA]_ss_ (ppb h^−1^)	k_GA_[O_3_]_ss_[GA]_ss_ (ppb h^−1^)	
1, 30	0.13	1.9	1.1	0.90	0.97
1, 60	0.025*	0.84	0.29	0.55	0.99
3, 30	0.14	3.2	2.3	0.99	0.97
3, 60	0.11	4.9	2.8	2.3	0.96

Note

The numbers in the column labeled *Condition* refer to the AER (h^−1^) and the ozone mixing ratio (ppb).

* See discussion of Condition 7 in ‘Limitations of the Study’*.*

The highest steady‐state mixing ratio of geranyl acetone (Table 2) occurred under Condition 3 (low AER, low O_3_), while the lowest occurred under Condition 7 (low AER, high O_3_). The primary and secondary reactions that generate geranyl acetone are together captured in the term “*k*_shirt_yield_GA_[O_3_]_ss”_ (see Equation (4)).

Air exchange rate and gas‐phase reactions with ozone are the sinks for geranyl acetone in the chamber. For Condition 3, these sinks are of similar magnitude; this is also the case for Condition 6. For Condition 7, removal via gas‐phase reaction occurs at about twice the rate of removal via ventilation; for Condition 5 the opposite is true. These reactions form further products, such as acetone, 6‐MHO, 4‐OPA, 4‐methyl‐8‐oxo‐4‐nonenal (4MON), and 4‐oxopentanoic (levulinic) acid. In our modeling, we focused on the ozonolysis of geranyl acetone in the gas‐phase to form 6‐MHO and 4‐OPA (Figure 1).

Table 5 shows steady‐state model parameters for 6‐MHO. The yield (Yield_6MHO_), which is that fraction of ozone removed by the shirt that produces 6‐MHO, has been adjusted to produce source‐to‐sink ratios near unity. The estimated yield for 6‐MHO from surface reactions was between 3.0% and 4.4% for Conditions 3, 5, and 6, while it was only 1.3% for Condition 7.

**Table 5:** Steady‐state model parameters for 6‐MHO

Condition AER, ozone (h^−1^, ppb)	Yield_6MHO_ (−)	f_6MHO_ (−)	Source terms		Sink terms		Source/sink ratio
			k_shirt_yield_6MHO_[O_3_]_ss_ (ppb h^−1^)	*k*_GA_f_6MHO_[O_3_]_ss_[GA]_ss_ (ppb h^−1^)	λ[6MHO]_ss_ (ppb h^−1^)	*k*_6MHO_[O_3_]_ss_[6MHO]_ss_ (ppb h^−1^)	
1, 30	0.044	0.3	0.65	0.27	0.65	0.27	1.00
1, 60	0.013*	0.3	0.44	0.17	0.32	0.30	0.99
3, 30	0.030	0.3	0.69	0.30	0.81	0.17	1.00
3, 60	0.040	0.3	1.8	0.70	1.8	0.73	1.01

Note

The numbers in the column labeled *Condition* refer to the AER (h^−1^) and the ozone mixing ratio (ppb).

* See discussion of Condition 7 in ‘Limitations of the Study’

The highest steady‐state mixing ratio of 6‐MHO (Table 2) occurred under Condition 3 (low AER, low O_3_), while the lowest occurred under Condition 4 (high AER, low O_3_). The production of 6‐MHO on the shirts (the term *k*_shirt_yield_6MHO_[O_3_]_ss_ in Equation (6)) constituted about ~70% percent of the total production of 6‐MHO inside the chamber. The gas‐phase reaction of geranyl acetone with ozone (the term *k*_GA_f_6MHO_[O_3_]_ss_[GA]_ss_) constituted the remaining ~30% of its production.

The sinks of 6‐MHO are air exchange and interaction with ozone in the gas‐phase. Air exchange accounted for 70%‐80% of the removal and dominated the approach of 6‐MHO mixing ratios to steady‐state for Conditions 3, 5, and 6. Reaction with ozone was responsible for the remaining 20%‐30% of the sinks. The two sink terms were of similar magnitude for Condition 7 (low AER, high O_3_). The reaction additionally forms secondary and tertiary products such as 4‐oxopentanal, 4‐oxopentanoic acids, acetone, and hydroxyacetone.

Table 6 shows steady‐state model parameters for 4‐OPA. The yield (Yield_4OPA_), which is that fraction of ozone removed by the shirt that produces 4‐OPA, has been adjusted to produce source‐to‐sink ratios near unity. The fitted yield for 4‐OPA varied among the conditions and was highest for Condition 6 (9.2%) and lowest for Condition 7 (3.8%).

**Table 6:** Steady‐state model parameters for 4‐OPA

Condition AER, ozone (h^−1^, ppb)	Yield_4OPA_ (−)	f_4OPA_ (−)	Source terms			Sink term	Source/sink ratio
			*k*_shrt_yield_4OPA_[O_3_]_ss_ (ppb h^−1^)	*k*_6MHO_[O_3_]_ss_[6MHO]_ss_ (ppb h^−1^)	*k*_GA_f_4OPA_[O_3_]_ss_[GA]_ss_ (ppb h^−1^)	λ[4OPA]_ss_ (ppb h^−1^)	
1, 30	0.066	0.7	0.98	0.27	0.63	1.9	1.00
1, 60	0.038*	0.7	1.3	0.30	0.39	2.0	1.00
3, 30	0.053	0.7	1.2	0.17	0.69	2.1	1.00
3, 60	0.092	0.7	4.1	0.73	1.6	6.5	1.00

Note

The numbers in the column labeled *Condition* refer to the AER (h^−1^) and the ozone mixing ratio (ppb).

* See discussion of Condition 7 in ‘Limitations of the Study’

The highest steady‐state mixing ratios of 4‐OPA (Table 2) occurred under Conditions 3 (low AER, low O_3_), 7 (low AER, high O_3_), and 6 (high AER, high O_3_), while the lowest occurred for Condition 5 (high AER, low O_3_). The production on the surfaces of the t‐shirts (the term k_shirt_yield_4OPA_[O_3_]_ss_ in Equation (8)) accounted for 50%‐65% of the total production rate. The gas‐phase reaction of 6‐MHO with ozone accounted for 10%‐15% (term k_6MHO_[O_3_]_ss_[6MHO]_ss_) and the gas‐phase reaction of geranyl acetone with ozone for 20%‐30% (term k_GA_f_4OPA_[O_3_]_ss_[GA]_ss_). The reported yield for Condition 3 may be slightly low because 4‐OPA mixing ratio has not reached steady‐state. Unlike 6‐MHO and geranyl acetone, 4‐OPA is removed only through ventilation; it does not have C‐C double bonds to further react with ozone. Thus, the rate of removal of 4‐OPA is determined by air exchange.

Summing up, geranyl acetone was formed entirely through ozone‐initiated reactions on the t‐shirts (with squalene and its unsaturated reaction products), while ~70% of 6‐MHO and 50%‐65% of 4‐OPA were formed through such surface reactions. The removal of 4‐OPA was entirely through ventilation, while the removal of 6‐MHO was 70%‐80% through ventilation. The relative contribution of ventilation and gas‐phase reactions to the removal of geranyl acetone varied with the Condition.

**Figure 5:**
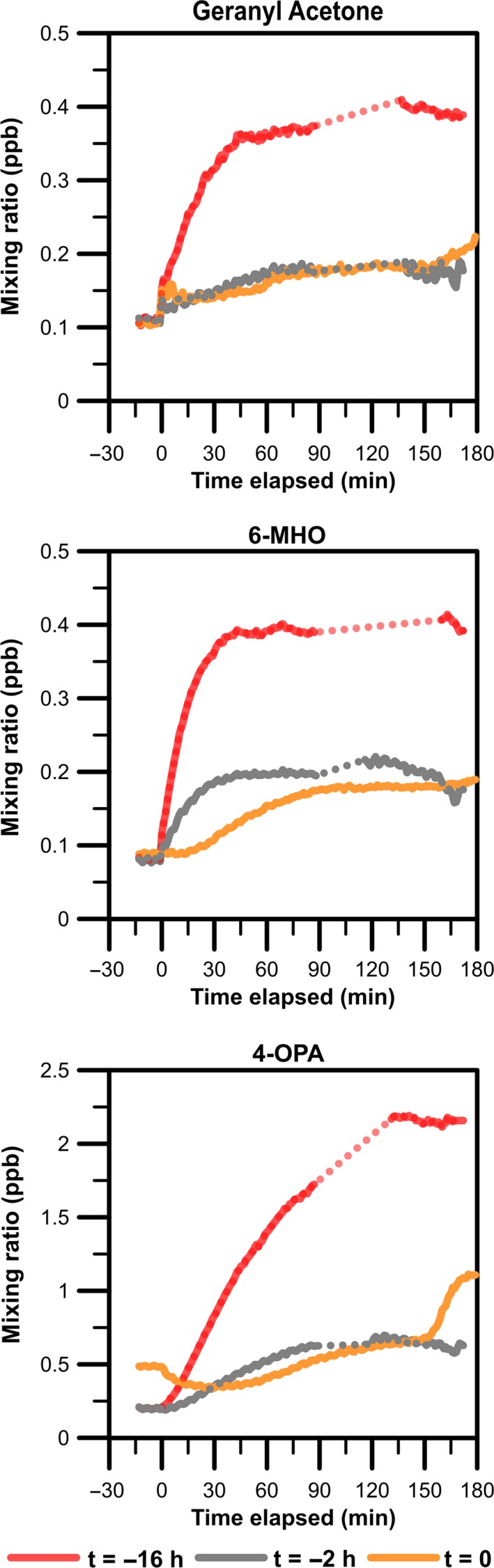
Time series of geranyl acetone, 6‐MHO, and 4‐OPA during experiments with intermittent ventilation (Conditions 8 and 9). Condition 7 (continuous ventilation) is shown for comparison. Time (t) is the time when ozone generation began relative to when the t‐shirts were placed in the chamber. Note that the concentrations are not corrected for background mixing ratios in the chamber. Background measurements were unavailable prior to Conditions 8 and 9 due to the nature of the experiments (intermittent ventilation: reduced AER and no ozone generation overnight)

Figure 5 shows the mixing ratios of geranyl acetone, 6‐MHO, and 4‐OPA over time for Conditions 7, 8, and 9. The steady‐state mixing ratios are presented in Table 2. By t = 180 minutes, geranyl acetone appears to have reached a semi‐steady‐state mixing ratio in Conditions 7 (ozone generation began at t = −16 hours) and 9 (t = −2 hours), but not in Condition 8 (t = 0 hours). The same appears to be true for 6‐MHO and 4‐OPA. However, the accelerated mixing ratio growth observed between 150 and 180 minutes for 4‐OPA and to a smaller extent for geranyl acetone was unexpected and may reflect chemistry taking place that is not accounted for in the current mechanistic model.

In Condition 8, the increase in 6‐MHO’s mixing ratio was not apparent until about 10 minutes after ozone and the t‐shirts were simultaneously introduced to the chamber. This is consistent with the findings of Wisthaler and Weschler,^13^ who observed a 30‐40 minutes delay in the increase of the oxygenated VOC after the start of ozone generation in an occupied simulated office. It is however unclear why a similar delay was not observed for geranyl acetone. In Condition 8, an elevated background mixing ratio was observed for 4‐OPA before the experiment commenced. This may reflect off‐gassing of 4‐OPA from the chamber walls during the night when the ventilation rate was reduced. When the ventilation was increased to 1 h^−1^ at the start of the experiment, the 4‐OPA mixing ratio initially decreased before an increase was observed.

Intermittent ventilation (reduced AER, no ozone generation the night before experiment) resulted in substantially lower steady‐state mixing ratios of geranyl acetone, 6‐MHO, and 4‐OPA. While in Condition 7 (t = −16 hours) the soiled t‐shirts were placed in an “ozone rich” and chamber, in Condition 8 (t = 0 hour) they were placed in an “ozone poor” chamber. Condition 9 (t = −2 hours) was, however, relatively “ozone rich”, with [O_3_] = 53 ppb at the time the t‐shirts were introduced, and we would anticipate steady‐state mixing ratios closer to those of Condition 7 than actually observed. Additionally, after a sufficiently long period, all three experiments would be anticipated to approach a similar steady‐state mixing ratios under otherwise identical final conditions (AER = 1 h^−1^, 60 ppb of ozone).

The steady‐state mixing ratios under intermittent conditions are somewhat sensitive to the mix of unsaturated products present on the surfaces of the t‐shirts and the chamber walls. The differences may be caused, in addition to the different starting ozone mixing ratios at the time the t‐shirts were placed in the chamber, by differences in soiling and differences in the extent to which skin lipids have been oxidized before the t‐shirts were placed in the chamber.

## 4 LIMITATIONS OF THE STUDY

Acetone is known to be a major product of squalene ozonolysis. However, we were unable to quantify it, since the instrument had poor response to this molecule.

The yields of formation for geranyl acetone, 6‐MHO, and 4‐OPA in the models were adjusted to achieve source‐to‐sink ratios close to unity. These yields were similar for Conditions 3, 5, and 6, while they were substantially lower for Condition 7. This latter condition occurred early in the campaign after a power outage. However, the humidity and temperature within the chamber was unaffected by the outage, and the mass calibration of the ToF‐CIMS did not change substantially. The total ion count was approximately 10% lower during Condition 7, and the ratio of water dimer to monomer was 20% lower. The signal normalized by the mixing ratio of the reagent ion partially takes this into account. Nonetheless, the accuracy of the measurements depends on many parameters. We decided to still report the data from Condition 7 since it provided valuable information on the dynamics of the product distribution within the chamber.

Although the measured values of k_chamber_ (0.16 h^−1^ and 0.15 h^−1^) were consistent with ozone loss rates (0.06‐0.35 h^−1^) previously reported^27^, ^28^, ^29^, ^30^ in empty stainless steel chambers, these values may represent reactivity of the normal chamber surfaces plus newly deposited reactants. They were determined under conditions in which there were likely freshly generated SVOC products sorbed on the chamber surfaces. The impact of such freshly generated SVOCs is anticipated to be small, since most of the products that partitioned to the chamber surfaces did not contain unsaturated carbon bonds and would not react with ozone.

While the use of a steady‐state model is justified for 6‐MHO and geranyl acetone, this is not always the case for 4‐OPA. For Condition 3, it is apparent that the mixing ratio of 4‐OPA is still slightly increasing during the final 40 minutes of measurement. In hindsight, it would have been valuable to have made measurements over a longer time period for this condition. Given that 4‐OPA was not at steady‐state, the results for 4‐OPA, Condition 3, should be viewed as rough approximations.

We acknowledge that the lack of replications is a major limitation of our study. We scheduled Condition 4 as a replicate for Condition 7 (1 AER/60 ppb O_3_). Unfortunately, due to analytical issues, no reliable measurements of oxygenated products were obtained from this experiment.

The sudden increase in the mixing ratios of geranyl acetone and 4‐OPA observed toward the end of the measurements in Condition 8 (Figure 5) remains unexplained.

## 5 CONCLUSIONS

Occupant‐related indoor chemistry is complex. In the case of unsaturated species such as geranyl acetone and 6‐MHO, ozone can both produce and consume a molecule. Steady‐state mixing ratios of squalene‐ozone reaction products are a result of an interplay between primary, secondary, and tertiary gas‐phase and surface reactions, as well as ventilation. In order to quantify the formation and removal processes, a series of mass balance equations was applied to measurement data obtained for a number of experimental conditions in a climate chamber.

When previously worn t‐shirts were in the chamber, more than 96% of the ozone removal was attributable to the combination of surface reactions and ventilation (about 40% at AER = 1 h^−1^ and 70% at AER = 3 h^−1^), while gas‐phase reactions accounted for less than 4% of the ozone removal. The ventilation strategies have only a marginal effect on the ozone removal rate by the t‐shirts, which is mass transport limited.

Geranyl acetone is produced only by ozone reactions occurring on the surface of the t‐shirts. Ventilation was responsible on average for about 50% of its removal from the gas‐phase, while the rest was attributable to further reactions with ozone. Approximately 70% of 6‐MHO was produced on the surfaces of the t‐shirts, while secondary gas‐phase reaction of ozone with geranyl acetone was responsible for the remaining formation. Air exchange was the dominant removal process for 6‐MHO (up to 80%), as opposed to further reactions with ozone in the gas‐phase. 4‐OPA is a secondary and tertiary product of squalene ozonolysis occurring on the surfaces of the t‐shirts. About 60% of 4‐OPA was produced through this pathway, 20%‐30% through gas‐phase reactions of geranyl acetone and the rest from gas‐phase reaction of 6‐MHO. 4‐OPA is only removed by ventilation.

Excluding Condition 7, the fitted formation yields (the fraction of ozone removed by the shirts that ultimately produced geranyl acetone, 6‐MHO, and 4‐OPA) were approximately 12%, 3%‐4%, and 5%‐10% for geranyl acetone, 6‐MHO, and 4‐OPA, respectively. The formation yield for GA is a mix of yields from surface chemistry and is relatively insensitive to ventilation rates. The overall yields of 6‐MHO and 4‐OPA are a mix of yields from surface chemistry and yields from gas‐phase chemistry and are moderately sensitive to ventilation rates. The yields are valuable inputs to predictive models.

Turning off the ventilation overnight or on weekends may lead to the accumulation of certain pollutants with indoor sources, but could also limit the extent to which ozone‐derived products are formed and will delay their generation when the ventilation is turned back on again. In applying such a practice, it should be recognized that ozone concentrations are often at their lowest daily values overnight. Preventing ozone‐initiated chemistry in indoor environments is better achieved by limiting indoor ozone concentration via filtration rather than adjusting ventilation rates.
