# Supplementary material for: Indoor ozone/human chemistry and ventilation strategies
Source: Indoor Air. 2019 Sep 15;29(6):913–25. doi: 10.1111/ina.12594 (PMC6856811; doi:10.1111/ina.12594)
Supplement: Supplementary file 1 [file INA-29-913-s001.pdf]

# Supporting Information

# Indoor ozone/human chemistry and ventilation strategies

Christian Mark Salvador[1], Gabriel Bekö[2], Charles J. Weschler[2,3], Glenn Morrison[4], Michael Le Breton[1,#], Mattias Hallquist[1], Lars Ekberg[5,6], Sarka Langer[6,7,*]

[1]University of Göteborg, Department of Chemistry and Molecular Biology, Atmospheric Sciences, SE-41296 Göteborg, Sweden

[2]International Centre for Indoor Environment and Energy, Department of Civil Engineering, Technical University of Denmark, Lyngby 2800, Denmark

[3]Environmental and Occupational Health Sciences Institute, Rutgers University, Piscataway, New Jersey 08854, United States

[4]Department of Environmental Sciences and Engineering, Gillings School of Global Public Health, The University of North Carolina at Chapel Hill, NC 27599, United States

[5]CIT Energy Management AB, SE-412 88 Göteborg, Sweden

[6]Chalmers University of Technology, Department of Architecture and Civil Engineering, Division of Building Services Engineering, Göteborg, Sweden

[7]IVL Swedish Environmental Research Institute, SE-400 14 Göteborg, Sweden

[#]Present address: Volvo Group Trucks and Technology Method and Technical Development, SE-417 15, Göteborg, Sweden

**Contents**

1. Calculation of concentration of analytes using mass loss approach
2. Calculation of $k_{sur}$
3. Long-term measurement of ozone removal on the soiled t-shirts
4. Steady state mixing ratios

**Figures and Tables**

Figure S1: Typical mass loss regression plot used to calculate permeation rate.

Figure S2. Long-term measurement of soiled t-shirts' ozone removal. Orange points: four t-shirts from Condition 7; grey points: four t-shirts from Condition 4; blue points: four t-shirts used in earlier experiments/Conditions (randomly selected).

Figure S3: Time series plots of 4-OPA, 4-MON, 4-MOD in different conditions of air exchange rate and ozone mixing ratio.

Figure S4: Derivative plots of the geranyl acetone, 6-MHO and 4-OPA.

Table S1: Calculated ozone removal rate ($\mathbf{k_{sur}}$).

## 1. Calculation of mixing rations of analytes using mass loss approach

Sensitivities of the VOCs were obtained by flowing nitrogen gas over a permeation source at a constant temperature. These permeation sources were small vials that contained the target VOCs and were capped with glass critical orifices. These vials were placed in a larger glass container, which are suspended over a water bath to maintain constant temperature. The rate of permeation was determined through gravimetric analysis by measuring the change of the mass of the vial over a period of time (as shown in Figure S1).

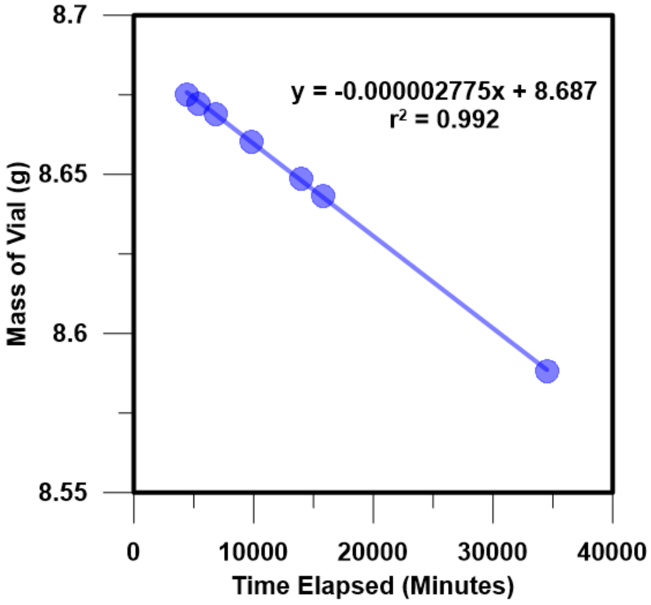


**Figure S1**. Typical mass loss regression plot used to calculate permeation rate

The sensitivity (cps ppb$^{-1}$) was subsequently calculated using equation S1:

$$\boldsymbol{Sensitivity\ (cps \cdot ppb^{-1})} = Signal\ (cps) \cdot \left(\frac{m \cdot Na}{FR \cdot MW \cdot 2.46 \times 10^{13}}\right)^{-1} \quad \text{(S1)}$$

where *m* is the slope of the regression analysis between elapsed time and mass of vial, *Na* is Avogadro's number, *FR* is the flow rate of the $N_2$ introduced to the CIMS in liter per minutes (LPM), and *Signal* is the average counts per seconds (cps) measured when the VOCs were introduced to the CIMS.

## 2. Calculation of $k_{sur}$

Values for $k_{sur}$ were calculated from the measured generation rates (ppb h$^{-1}$) for ozone injected into the chamber, the steady state mixing ratio of ozone at each condition, and the corresponding value of the Air Exchange Rate (AER).

$$k_{sur} = \text{(generation rate/steady state mixing ratio) - AER}$$

**Table S1**. Calculated ozone removal rate ($k_{sur}$) from all the surfaces (chamber and soiled shirts) per experiment/condition.

| Date of experiment | Condition | AER ($h^{-1}$) | Ozone true emission rate (ppb $h^{-1}$) | Ozone steady-state mixing ratio (ppb) | $k_{sur}$ ($h^{-1}$) |
|---|---|---|---|---|---|
| October 9 | 7 | 1 | 62 | 28 | 1.23 |
| October 23 | 5 | 3 | 83 | 19 | 1.38 |
| October 25 | 6 | 3 | 161 | 37 | 1.39 |
| October 26 | 3 | 1 | 31 | 12 | 1.50 |

The average value of $k_{sur}$ from conditions 3, 7, 5 and 6 of (1.37 ± 0.11) $h^{-1}$ was used to calculate $k_{shirt}$, subsequently used in the mass balance models in the article (Equations 2, 4, 6 and 8). We used the same relative uncertainty for calculating the uncertainty of $k_{shirt}$.

## 3. Long-term measurement of ozone removal on the soiled t-shirts

Four soiled t-shirts from all previously measured conditions were randomly selected and placed again in the chamber. The ozone mixing ratio in the chamber was followed for about 45 hours. The results were compared with those obtained during approximately 10 hours for Conditions 4 and 7 (AER = 1 $h^{-1}$, 60 ppb of target ozone; Figure S2).

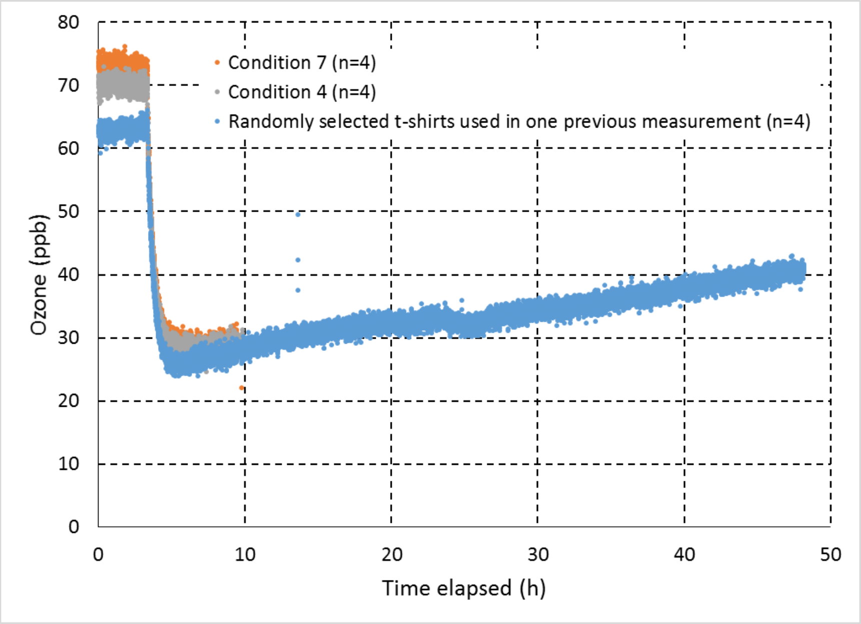


**Figure S2.** Long-term measurement of soiled t-shirts' ozone removal. Orange points: four t-shirts from Condition 7; grey points: four t-shirts from Condition 4; blue points: four t-shirts used in earlier experiments/Conditions (randomly selected).

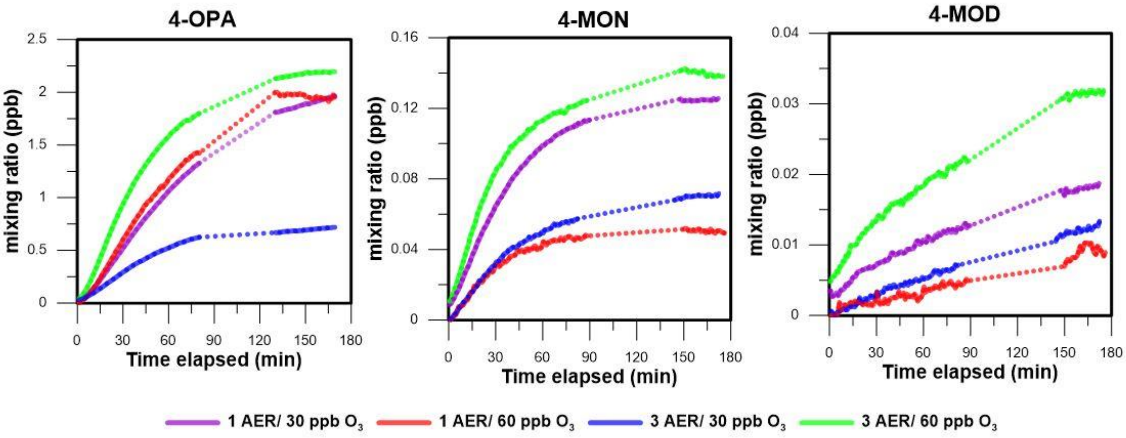


**Figure S3**. Time series plots of 4-OPA, 4-MON, 4-MOD at different conditions of air exchange rate and ozone mixing ratio.

Note that each plot has different y-axis limits due to different maximum values of the steady state mixing ratios.  4-OPA can reach as much as 2.0 ppb while the 4-MON and 4-MOD only reached ~ 0.14 ppb and ~ 0.03 ppb, respectively.

## 4. Steady state mixing ratios

The steady state mixing ratio for the oxygenated VOCs is the value of the mixing ratio when the change of mixing ratio over the change of time is zero ($\Delta C/\Delta t \rightarrow 0$). Figure S4 shows derivative plots, $\Delta C/\Delta t$, for geranyl acetone, 6-MHO and 4-OPA for the different AERs and ozone mixing ratios. These plots are presented primarily to show the validity of the steady-state assumption, that is the steady-state mixing ratios used in the mass balance models (Equations 2, 4, 6 and 8).

Geranyl acetone and 6-MHO were already at steady state after the first 50 minutes of the experiments, while for 4-OPA, the steady-state values were reached (or approached) during the last 40 minutes of the experiments.

The plots also show the reaction dynamics of these compounds. Geranyl acetone and 6-MHO were formed almost immediately after placing the t-shirts in the chamber. Their maximum formation rate was reached during the first couple of minutes of the experiment, which is consistent with their formation through primary and secondary reactions of squalene with ozone. The maximum formation rate for 4-OPA was reached approximately 20 minutes after the beginning of the experiment, which is consistent with 4-OPA being formed in secondary and tertiary squalene/ozone reactions.

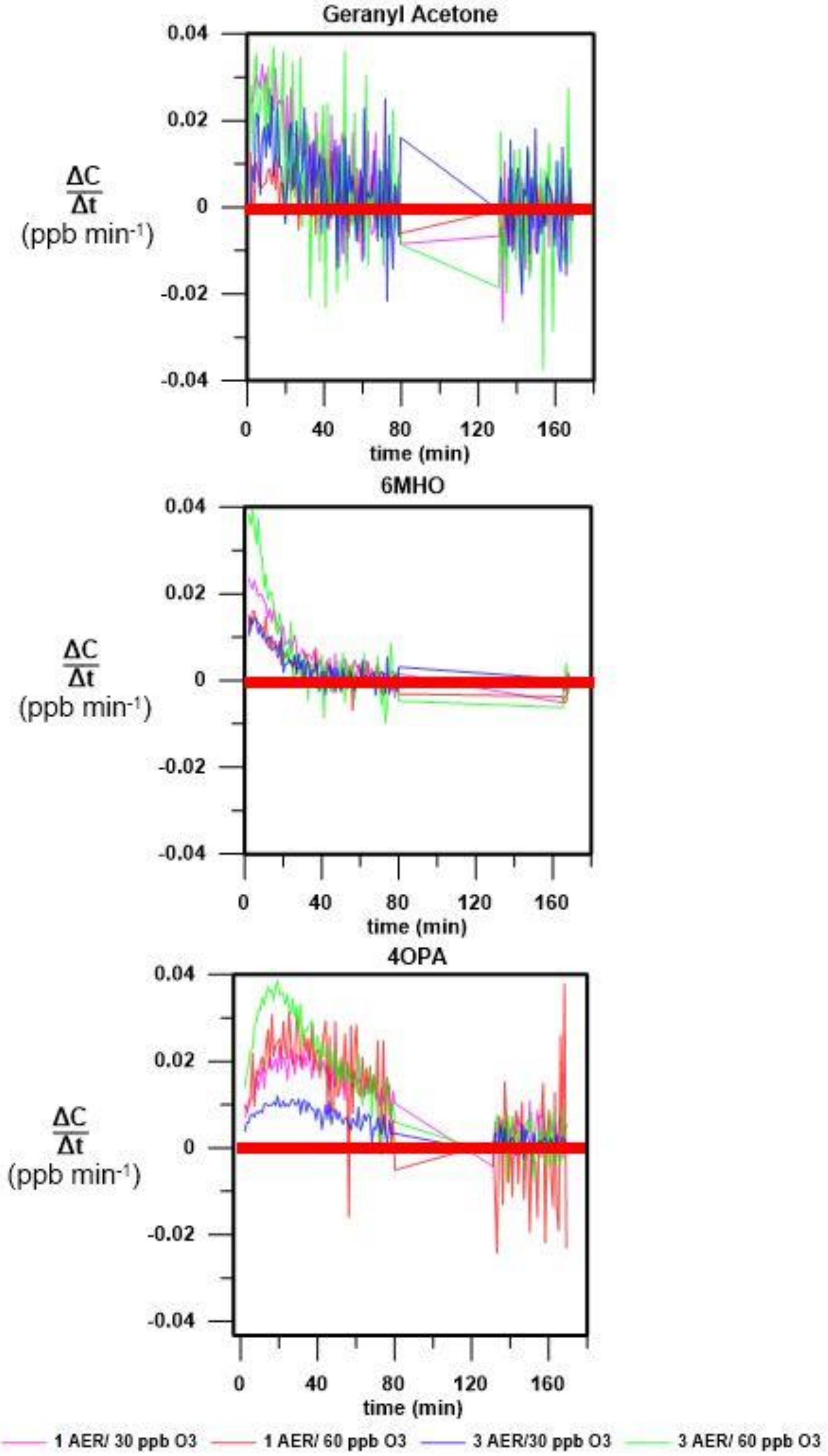


**Figure S4**. Derivative plots of geranyl acetone, 6-MHO and 4-OPA. The thick red line indicates the steady-state condition.
